# Development of a quantitative evaluation system for visuo-motor control in three-dimensional virtual reality space

**DOI:** 10.1038/s41598-018-31758-y

**Published:** 2018-09-07

**Authors:** Woong Choi, Jongho Lee, Naoki Yanagihara, Liang Li, Jaehyo Kim

**Affiliations:** 1 Department of Information and Computer Engineering, National Institute of Technology, Gunma College, Maebashi, 371-8530 Japan; 2 Department of clinical engineering, Komatsu University, Komatsu, 923-8511 Japan; 3 Graduate School of Systems and Information Engineering Department of Computer Science, University of Tsukuba, Tsukuba, 305-8577 Japan; 4 College of Information Science and Engineering, Ristumeikan University, Kusatsu, 525-8577 Japan; 5 Department of Mechanical and Control Engineering, Handong Global University, Pohang, 37554 Republic of Korea

## Abstract

The process of learning a human’s movement and motor control mechanisms by watching and mimicking human motions was based on visuo-motor control in three dimensional space. However, previous studies regarding the visuo-motor control in three dimensional space have focused on analyzing the tracking tasks along one-dimensional lines or two-dimensional planes using single or multi-joint movements. Therefore, in this study, we developed a new system to quantitatively evaluate visuo-motor control in three-dimensional space based on virtual reality (VR) environment. Our proposed system is designed to analyze circular tracking movements on frontal and sagittal planes in VR space with millimeter level accuracy. In particular, we compared the circular tracking movements under monocular and binocular vision conditions. The results showed that the accuracy of circular tracking movements drops approximately 4.5 times in monocular vision than that in binocular vision on both frontal and sagittal planes. We also found that significant difference can be observed between frontal and sagittal planes for only the accuracy of X-axis in both monocular and binocular visions.

## Introduction

Visually-guided tracking movements are important in visuo-motor control tasks such as imitation learning of using tools, dance, and sports. Studies regarding the visually-guided tracking movements have focused on tracking tasks along one-dimensional lines or two-dimensional planes based on multi-joint movements in three-dimensional space^1–10^ examined visuo-motor tracking of one-dimensional sinusoidal visual targets in manual tracking tasks involved humans and monkeys using multi-joint arm movements. As a result, visuo-motor control varied according to the presence of periodicity in target trajectory. In addition, Beppu *et al*.^4,5^ analyzed motor controls along ramp trajectories of patients with cerebellar ataxia and normal subjects in one degree-of-freedom elbow movement tracking tasks. They extracted parameters which could quantitatively evaluate disease severity. Previous studies showed that in these tracking movement tasks, visuo-motor control and/or evaluation parameters varied according to the dimension of the trajectories (i.e. trajectory on a one-dimensional line or trajectory on a two-dimensional plane). Therefore, the visuo-motor control and/or evaluation parameters in the tracking tasks should be determined by the dimension of the trajectories^11^.

Circular tracking movements are similar to one-dimensional sinusoidal tracking movements in the sense of periodicity. However, different from one-dimensional tracking movements, circular tracking tasks allow continues movements with a uniform velocity on two-dimensional planes^7–10,12–14^. Studies examined two-dimensional visually-guided tracking movements by carrying out wrist and hand tracking movement tasks using pen tablets, two-dimensional tracers (i.g. computer mouses), and two-dimensional manipulanda. In other words, they only measured and analyzed three dimensionally moveable wrist and hand tracking movements under two-dimensional visual guidance with two-dimensional tracing devices. However, a human visuo-motor control system performs three-dimensional movements by recognizing and adapting changes in three-dimensional environments. How to quantitatively evaluate and analyze human’s natural three-dimensional tracking movements is still an open question.

Recently, with the development of virtual reality (VR) technology, increasing researches, such as wayfinding^15^, proprioception^16^, and visuo-motor adaptation^17^, have been carried out in three-dimensional virtual space. In particular, Anglin *et al*. investigated the mechanisms of visuo-motor adaptation in head-mounted virtual reality versus conventional training^17^. They constructed a three-dimensional experimental environment that allowed two-dimensional circular tracking movements. Target error of angle between hand and a target which rotated along the obit was analyzed. A digitalized pen with a tablet was used as the tracer to measure the hand movements. However, they has not been adopted and analyzed the circular tracking movements in a three-dimensional VR environment.

**Figure 1:**
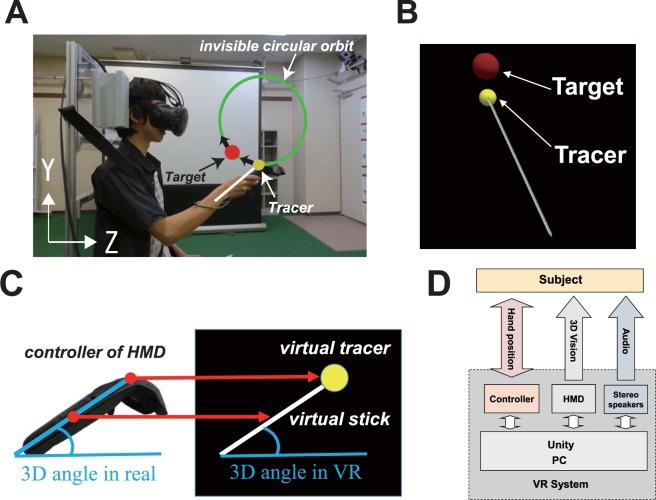
Experiment setup. (**A**) A real human user using the proposed system during the experiment. (**B**) Display on the HMD during experiment task. In VR space, Subjects used the tracer to track the target, which moves circularly in the clockwise direction. (**C**) Relationship between the controller of HMD and the virtual tracer and virtual stick in VR. (**D**) Configuration of the virtual reality system.

Therefore, in this study, we developed an evaluation system for three-dimensional visuo-motor control in VR environment (see Fig. 1). In particular, we adopted circular tracking task in three-dimensional VR space with the following requirements. (1) Movement tasks can be performed in a VR space with a seamless three-dimensional stereoscopic vision, which resemble seated manual movements in daily life. (2) A virtual target is displayed within arm’s reach. The target can be tracked by arm movements in a three-dimensional coordinate. (3) Tracer’s movements can be precisely measured and quantitatively evaluated.

In addition, to confirm the effectiveness of the developed system, we analyzed two types of target tracking movements: circular tracking movement on frontal plane to the body, and circular tracking movement on sagittal plane to the body. Furthermore, we compared the visuo-motor controls in three-dimensional circular tracking movements between monocular vision and binocular vision conditions.

## Results

**Figure 2:**
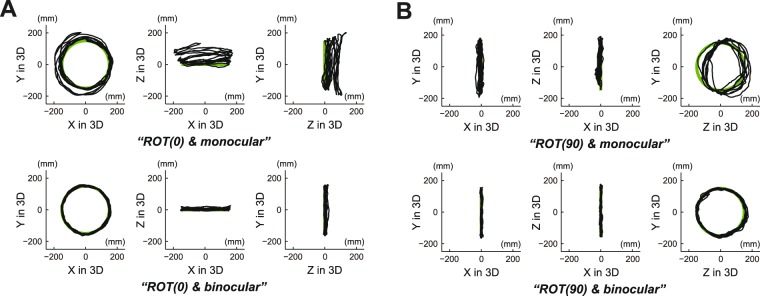
A typical example of circular tracking movement which was performed in 3D VR space. (**A**) Circular tracking movement on the frontal plane (*ROT(0)* condition). (**B**) Circular tracking movement on the sagittal plane (*ROT(90)* condition). For A and B, upper graphs are circular tracking movements under the monocular vision and lower graphs are circular tracking movements under the binocular vision. For all traces, three graphs show target paths (green line) and tracer paths (black line) on the front (left graph), the upper (center graph), and the side (right graph) for subjects’ eyes in experiment task.

Figure 2 shows a typical example of circular tracking movement which was performed in 3D VR space. Figure 2A represents circular tracking movements on the frontal plane (see *ROT(0)* condition in the *Procedure* section of *Methods*), while Fig. 2B indicates those on the sagittal plane (see *ROT(90)* condition in the *Procedure* section of *Methods*). Furthermore, upper graphs in Fig. 2A and B show the movements performed under monocular vision condition, while lower graphs show those performed under binocular vision condition. As shown in Fig. 2, tracking accuracy with binocular vision was significantly superior to that with monocular vision on both the frontal plane and the sagittal plane.

**Figure 3:**
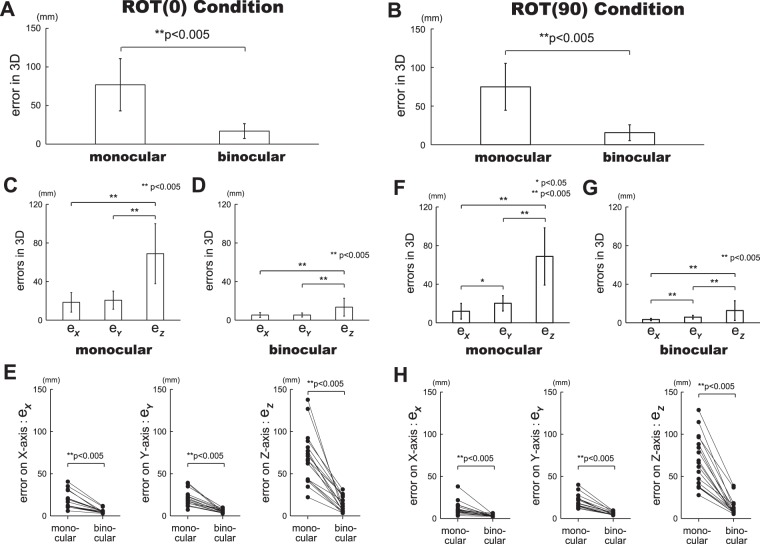
Evaluation of circular tracking movement based on error in VR space. (**A**) Difference between monocular and binocular visions in circular tracking movements on the frontal plane (*ROT(0)* condition). (**B**) Difference between monocular and binocular visions in circular tracking movements on the sagittal plane (*ROT(90)* condition). For A and B, note significant difference in the errors between the monocular vision and the binocular vision. (**C**) Analysis results in monocular vision (*ROT(0)* condition). (**D**) Analysis results in binocular vision (*ROT(0)* condition). (**E**) Comparison between monocular and binocular visions (*ROT(0)* condition). (**F**) Analysis results in monocular vision (*ROT(90)* condition). (**G**) Analysis results in binocular vision (*ROT(90)* condition). (**H**) Comparison between monocular and binocular visions (*ROT(90)* condition).

In this study, we quantitatively evaluated visuo-motor control in circular tracking movements by analyzing distance errors in the three-dimensional VR space between the target and the tracer. Figure 3A and B represent the errors in monocular and binocular visions on the two planes. On the frontal plane (Fig. 3A), significant difference (p = 8.7 × 10^−7^) can be observed between the errors in monocular vision (mean error = 76.9, SD = ±33.9) and binocular vision (mean error = 16.9, SD = ±9.6).

On the other hand, for the sagittal plane (Fig. 3B), significant difference (p = 3.9 × 10^−8^) can also be observed between the errors in monocular vision (mean error = 75.0, SD = ±30.5) and binocular vision (mean error = 15.6, SD = ±10.2). The accuracy of visuo-motor control in circular tracking movements drops approximately 4.5 times in binocular vision than that in monocular vision on both planes.

We also investigated the accuracy of visuo-motor control in circular tracking movements based on each axis of three-dimensional coordinate (Fig. 3C–H). Figure 3C–E represent the errors of each axis on the frontal plane, while Fig. 3F–H indicates those on the sagittal plane. For the binocular vision on the frontal plane (Fig. 3D), the errors on Z-axis (*e*_*Z*_ in Fig. 3D, mean value = 13.4) were significantly greater than the errors on X-axis (*e*_*X*_ in Fig. 3D, mean value = 5.2) and Y-axis (*e*_*Y*_ in Fig. 3D, mean value = 5.3) directions (p = 0.0003 for *e*_*X*_,and *e*_*Z*_; p = 0.0004 for *e*_*Y*_, and *e*_*Z*_). However, in the monocular vision (Fig. 3C), significant greater the errors on X-axis (*e*_*X*_ in Fig. 3C, mean value = 18.4), Y-axis (*e*_*Y*_ in Fig. 3C, mean value = 20.7), and Z-axis (*e*_*Z*_ in Fig. 3C, mean value = 68.8) directions than those in binocular vision can be observed (see Fig. 3E; p = 7.5 × 10^−6^ for *e*_*X*_, p = 1.8 × 10^−6^ for *e*_*Y*_, p = 1.1 × 10^−6^ for *e*_*Z*_).

On the other hand, for the binocular vision on the sagittal plane (Fig. 3G), the errors on Y-axis (*e*_*Y*_ in Fig. 3G, mean value = 5.8) were significantly greater than those on X-axis direction (*e*_*X*_ in Fig. 3G, mean value = 3.3; p = 1.0 × 10^−7^). The errors on Z-axis (*e*_*Z*_ in Fig. 3G, mean value = 12.6) were significantly greater than the errors on X-axis and Y-axis directions (p = 0.0009 for *e*_*X*_, and *e*_*Z*_; p = 0.0049 for *e*_*Y*_, and *e*_*Z*_). In the monocular vision (Fig. 3F), significant greater errors on X-axis (*e*_*X*_ in Fig. 3F, mean value = 11.9), Y-axis (*e*_*Y*_ in Fig. 3F, mean value = 20.1), and Z-axis (*e*_*Z*_ in Fig. 3F, mean value = 68.7) directions than those in the binocular vision can be observed (see Fig. 3H; p = 0.0004 for *e*_*X*_, p = 1.7 × 10^−7^ for *e*_*Y*_, p = 6.0 × 10^−8^ for *e*_*Z*_).

**Figure 4:**
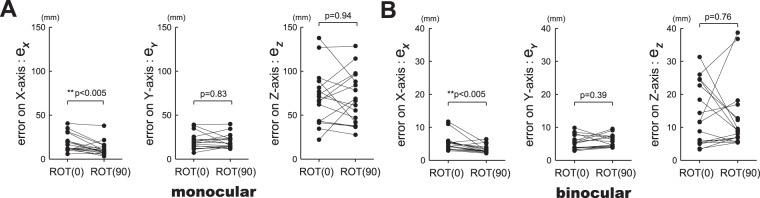
Comparison between the circular tracking movements on frontal plane (*ROT(0)*) and the circular tracking movement on sagittal plane (*ROT(90)*) based on the error on each axis of three-dimensional coordinate. (**A**) Analysis results in monocular vision. (**B**) Analysis results in binocular vision. Note significant difference on only X-axis direction between two tracking movement on different plane for both monocular and binocular visions.

Figure 4 demonstrates the difference between frontal plane (*ROT(0)*) and sagittal plane (*ROT(90)*) in terms of the error of each axis of three-dimensional coordinate. No significant difference was observed between frontal and sagittal planes for Y-axis (*e*_*Y*_ in Fig. 4) and Z-axis (*e*_*Z*_ in Fig. 4) directions, regardless of the vision condition. Whereas, on X-axis direction, significantly greater errors can be observed in frontal plane than those in sagittal plane for both monocular and binocular visions (p = 0.0049 for monocular vision; p = 0.0048 for binocular vision).

## Discussion

In this study, we proposed a system which enables us to quantitatively evaluate visuo-motor control in three-dimensional space based on target tracking in VR environment. Specifically, the proposed system enabled us to perform three-dimensional circular tracking movements in VR space with millimeter level accuracy. It is confirmed that three-dimensional visuo-motor control under monocular vision and binocular vision conditions could be analyzed clearly and quantitatively (see Figs 3 and 4). The results indicate that the accuracy of circular tracking movements drops approximately 4.5 times in monocular vision than that in binocular vision on both frontal and sagittal planes. We also compared the visuo-motor control between circular tracking movements on frontal (*ROT(0)* condition) and sagittal (*ROT(90)* condition) planes (see Fig. 4). As a result, significant difference can be observed between frontal and sagittal planes for only the accuracy of X-axis in both monocular and binocular visions.

In the following discussion, we will discuss about next two issues: (1) differences in three-dimensional visuo-motor control under monocular vision and binocular vision conditions; (2) possible applications of the proposed system.

### Differences in three-dimensional visuo-motor control under monocular vision and binocular vision conditions

Studies on visuo-motor control under different vision conditions have been reported mainly on reaching and grasping arm movements^18–21^. As a common result, because of perceptual uncertainty (e.g. precision of proper stimulus cue is low in monocular vision), it required longer time to make a movement. In contrast, velocity and the accuracy of hand movement in binocular vision is faster than that in monocular vision. As reported by Melmoth *et al*.^18^, in real environment, the accuracy of reaching and grasping movements in binocular vision had 2.5 to 3 times advantage over that in monocular vision. Our study obtained a similar result, in which the accuracy of circular tracking movements in binocular vision showed approximately 4.5 times advantage over that in monocular vision on both frontal and sagittal planes in three-dimensional VR environment (see Fig. 3A and B). In other words, our study not only confirmed that in VR environment tracking accuracy in binocular vision is superior to monocular vision, but also showed that the parameter precision in the three-dimensional VR environment provided by the proposed system was similar to that in the real environment. Furthermore, our results agree with Anglin(2017)’s results^17^. However, we carried out the experiments in a three-dimensional environment directly, instead of reproducing a two-dimensional circular movement experiment in a three-dimensional environment.

In this study, more significant decrement in visuo-motor control along Z-axis can be confirmed than that along X and Y axes (see Fig. 3C–H). This result agrees with McKee(2010)’s^22^ study that in the isolated setting, binocular depth thresholds for objects presented in a real environment were greatly superior to the monocular thresholds by as much as a factor of 18. However, in our study, difference in visuo-motor control along Z-axis on both frontal and sagittal planes has not been observed in neither monocular vision nor binocular vision (see Fig. 4). In real world, cues of occlusion and object size were used in depth perception. This is because the proposed system provided depth cues by presenting occlusion information of the target and the tracer^23^.

In this study, a similar increasing trend can be observed in the errors along X, Y, and Z axes between frontal and sagittal planes in both monocular and binocular visions (see Fig. 4). This result agrees with Haggard’s report that within a nearby arm reaching distance, positional information can be perceived with an accuracy of millimeter level^24^. In this study, we could examine the differences in three-dimensional position perception and visuo-motor control between monocular and binocular visions with millimeter level accuracy. We found that within an arm reaching distance, positional information of the target and the tracer can be more accurately perceived in binocular vision than that in monocular vision (see Fig. 4). Servos(1992) reported that perceived distance and perceived size in monocular vision have reduced to 86% and 81% respectively than in binocular vision^25^. Our result confirmed the differences in perception between the two visions in a three- dimensional VR environment. In addition, significant difference can be observed along X-axis between frontal and sagittal planes. It indicates that different control parameters and strategies were adopted in circular tracking movements on the two planes.

In previous studies, for one or two dimensionally moving targets, the control strategies and major control parameters for tracking movements vary whether the trajectory of target is random or consistent^3,9,14^. It was reported that the rate of reliance on the two strategies and the main control parameters were determined by the motion range (i.e. degree of freedom), speed, and the presence of visual information of the target^1–8,11,12,14^. The proposed system enables us to examine control strategies and control parameters with different variables (trajectory type, target speed, target/tracer visibility, etc.) for target-tracking in three-dimensional space. In future, more three-dimensional movements will be examined from the viewpoint of motor control based on not only spatial parameter like the 3D error of this study but also temporal parameters like theta and omega errors of our previous study^7^.

### Future application of the proposed system

In this study, we proposed a VR system that enables quantitative evaluation of visuo-motor control in three-dimensional tracking movements. Conventional studies on tracking movements mainly carried out using linear or planar stimuli and measurement devices^1–10,12–14^. However, analysis of three-dimensional multi-joint movements based on data obtained by one and two-dimensional devices are insufficient. Our system allows the subjects to perform three degree-of-freedom visuo-motor tracking movements in an immersive three-dimensional VR environment. Various movements can be measured and analyzed with a high degree of accuracy using the proposed system. In future work, our proposed system can be used for the evaluation of illness seriousness and rehabilitation effectiveness for patients with hemiplegic upper limb. It can be also applied to studies on perception of spatial neglect patients^26^.

Furthermore, similar to eye-hand coordination, the proposed system constructed a VR space where the user can explore the environment based on multisensory integration. In other words, the proposed system evaluates perception in visual space and motor space by tracking a circular moving target with a tracer in a three-dimensional VR environment. In particular, if an object is displayed instead of the subject’s own hand in the VR environment, the subject may experience an extension of their body ownership towards the object, known as the rubber hand illusion (RHI), by performing active movements^27^. In our system, similar to Iriki(2001)’s study, the subject’s body image is considered to be extended to the virtual tracer^28,29^. It is reported that the RHI is stronger when the rubber hand is located at the position of one’s real hand^30^. Furthermore, it has to be noted that efference copy^31^ and the sensory feedback should be coincided in time to evoke the sense of agency. Therefore, our system displays a virtual tracer to indicate the hand position and movement for gaining the body ownership and sense of agency. In this study, the virtual tracer was spatially and temporally synchronized to the movement of the subject’s hand. In future work, various perception evaluations in either realistic or manipulated conditions can be carried out using the proposed system.

The proposed system can be integrated with a haptic device with force feedback. Thus, not only the visuo-motor control, but also proprioception can be quantitatively evaluated^32^. Furthermore, spatial and/or force JND of the hand-arm system can be considered as one of the perception evaluation parameters in our future experiments^33,34^. For example, it can be used for quantitative evaluation in the development of rehabilitation system for stroke patients.

As shown in Table 1, in previous studies on target tracking in one-dimensional or two-dimensional spaces, the target size was set to the same or two to five times greater than the tracer size. In other words, in early studies with normal controls and patients with cerebellar ataxia or sensory ataxia, the target size was set to two to three times of the tracer size to analyze the effects of target speed and visual information in tracking movements^3–5,35–37^. In our previous studies^38,39^, to ensure patients with cerebellar ataxia can perform stable tracking movements in two-dimensional space, we set the target size five times greater than the tracer size. On the other hand, in the studies on target tracking strategies regarding feedback and/or feedforward control in one-dimensional or two-dimensional spaces^36,40,41^, the target size was set to the same as the tracer size. In this study, we also aimed to quantitatively analyze the target tracking strategies using the proposed system for visuo-motor control. However, our task was a more difficult one which was carried out in a three-dimensional space. Thus, we set the target (1.5 cm) 1.5 times bigger than the tracer (1.0 cm). The proposed system can be also used for analyzing target tracking movements of patients with cerebellar ataxia as well as normal controls. In this case, the target should be set to a bigger size as in our previous studies^38,39^. Furthermore, to investigate the effect of target size in tracking movements on three-dimensional space is one of our future works.

**Table 1:** Previous studies related with target size and tracer size: diameter on the display.

Previous studies	Target size	Tracer size
Nagaoka and Tanaka (*Neuroscience Letters*) 1981^35^	33 mm	11 mm
Beppu *et al*. (*Brain*) 1984^4^ (*Brain*) 1987^5^	33 mm	11 mm
Miall *et al*. (*Journal of Motor Behavior*) 1993^3^	2 mm × 4 mm (rectangle)	1 mm × 2 mm (rectangle)
Foulkes and Miall (*Exp. Brain Res*.) 2000^36^	4 mm ×× 4 mm (square)	4 mm × 4 mm (square)
Reed *et al*. (*Neuroscience Letters*) 2003^37^	12 × 12 pixel (square)	6 × 6 pixel (square)
Hayashi *et al*. (*Artif Life Robotics*) 2009^40^	6 mm	6 mm
Ao *et al*. (*Plos One*) 2015^41^	1 cm × 3 cm (slider)	1 cm × 3 cm (slider)
Lee *et al*. (*Cerebellum*) 2012^38^ (*PLoS One*) 2015^39^	10 mm	2 mm

## Methods

### Subjects

Seventeen subjects (17 males) with mean age 20.12 ± 0.6 (SD) participated in the experiments. All subjects had a normal or corrected-to-normal vision and were right-handed. None had previously participated in similar studies. All subjects gave written informed consents prior to their participation. Informed consent has been obtained for publication of Fig. 1A. All experiments were conducted in accordance with relevant guidelines and regulations. The protocol was approved by the ethics committees of National Institute of Technology, Gunma College.

### Configuration of the proposed system

Figure 1D shows the configuration of the system used in the experiments. In order to build a system allows quantitative evaluation of visuo-motor control in VR space, the system has to have following functions: (1) immersive 3D VR environment, (2) real-time tracking and recording of arm movement, (3) real-time rendering of movement-synchronized tracer in the VR environment. For achieve such a system, we used Unity 3D to build the 3D VR environment and used HTC Vive for the immersive display of it.

The VR environment which provides three-dimensional computer graphics (CG) and surrounding sound were built with Unity. Our experiments were performed on a PC with the following specifications: Intel i7-6700 CPU, 8GB of RAM, and NVIDIA GeForce GTX1070 GPU. Stereoscopic 3D CG was displayed on an HTC Vive HMD (resolution: 2,160 × 1,200; field of view: 110°; refresh rate: 90 Hz). The HTC Vive HMD provides us a hand-held controller which tracks, records and feeds back its spatial coordinate in real-time.

The HMD’s Lighthouse tracking system^42^ tracks the position of a controller with a position precision of 2 mm. We also measured the spatial error of the system using two experiments (three times for each). (1) Move the controller along x, y, and z axes 40 cm guided by a ruler, and compare with the corresponding positions of the virtual tracer. The position errors of the controller along x, y, and z axes were 0.43 ± 1.55 mm, 0.60 ± 0.60 mm, and 0.97 ± 1.02 mm, respectively. (2) Construct a cube with side length 40 cm in the virtual environment, and measure the position of the virtual tracer by the controller. The position errors along x, y, and z axes were 0.87 ± 0.47 mm, 0.56 ± 1.0 mm, and 0.27 ± 0.55 mm, respectively. The average frame rate of our proposed system in three trials of our experiments were 90.1 ± 0.133 FPS. The resolution of the HMD (HTC Vive) was 2160 × 1200 with a frame rate of 90 FPS. It indicates that the system delay per one frame is no greater than 11.1 msec (1/90 sec).

In our experiments, the controller was held by subjects with their right hands. The coordinates of the tip of the controller were tracked and recorded. The sounds in the experiment were played by a 3D sound speaker (Dr. Three 3D-02, http://www.dr-three.com/products/m3d02.html).

We used the library “SteamVR plugin for Unity v1.1.1” in the development. This library allows us to set several parameters regarding camera, projection method, field of view, and clipping planes. Especially, for the binocular disparity, we set the parameter to Camera(head) in the CameraRigPrefab function to attach the camera to the HMD. Consequently, we confirmed that the average errors of display and tracking were less than 1 mm, which ensured a suitable accuracy for our experiment.

### Experimental setup

The system allowed subjects to perform a visually-guided tracking task in a 3D VR environment (Fig. 1). In particular, considering the difficulty of tracking task in 3D space, we set the size of the target 1.5 times bigger that the tracer in this study. In other words, as shown in Fig. 1B, the target was a virtual red ball with a radius of 1.5 cm. Instead of subjects’ own hands or the HMD’s controller, they were shown a virtual stick. A tracer, which was a virtual yellow ball with a radius of 1 cm, was placed at the tip of the stick.

As shown in Fig. 1A, the tip of the controller was associated with the yellow virtual tracer. The subjects holds the handle of the controller during the experiment. The direction of the controller was synchronized with that of the virtual stick (Fig. 1C). In other words, the position of the tracer was synchronized with the subjects’ movements. Furthermore, since the red target ball was rendered transparently, the yellow tracer ball was visible even inside the red target ball. In the experiment, subjects were asked to track the target with the tracer in a 3D VR environment. The target moved constantly along an invisible circular orbit with a radius of 15 cm.

The circular orbit of the target can be defined as follows:

$$\begin{array}{rcl}P({P}_{x},{P}_{y},{P}_{z}) & = & (radius\times sin(\frac{\pi \theta }{180.0})\times cos(\frac{\pi r}{180.0}),radius\\  &  & \times cos(\frac{\pi \theta }{180.0}),radius\times sin(\frac{\pi \theta }{180.0})\times sin(\frac{\pi r}{180.0})),\end{array}$$

where, *P*(*P*_*x*_, *P*_*y*_, *P*_*z*_) is the coordinate of the target and *P*_*x*_, *P*_*y*_, *P*_*z*_ indicate the coordinates for x-axis [m], y-axis [m], z-axis [m] of the target. *radius* is the radius of the circular movement[m]. *r* is the rotation angle along y axis $(0.0^\circ \le r < 360.0^\circ )$. *θ* is the angle of circular movement $(0.0^\circ \le \theta  < 360.0^\circ )$. The axis of rotation was set to several orientations according to the requirements of the experiment.

**Figure 5:**
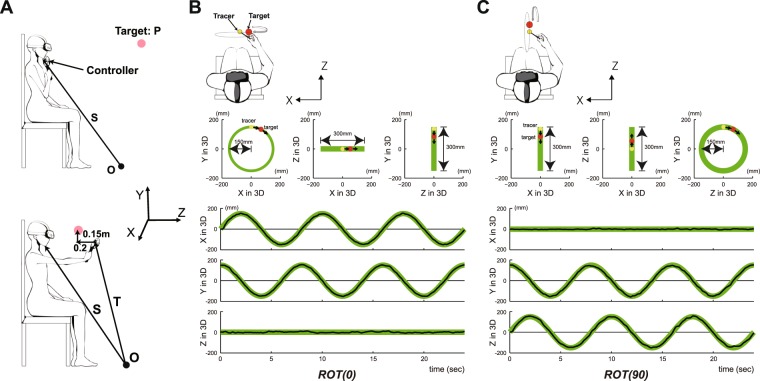
Experiment procedure. (**A**) In order to initialize the position of Y-axis of the target, the position of the chin of the subject is measured. In order to initialize the positions of X-axis and Z-axis of the target, the system measures the stretched position for the subject’s hand. (**B**) Procedure of circular tracking experiments on the frontal planes (*ROT(0) condition*). Top inset illustrates circular tracking experiment on the frontal planes of the body on the HMD (*ROT(0)* in Figs 2–5). (**C**) Procedure of circular tracking experiments on the sagittal planes (*ROT(90) condition*). Top inset illustrates circular tracking experiments on the sagittal planes of the body on the HMD (*ROT(90)* in Figs 2–5). For B and C, green lines indicate target path in three-dimensional VR space. In other words, three graphs in second trace demonstrate the target path on the front (left graph), the upper (center graph), and the side (right graph) for subjects’ eyes in experiment task. However, the target path was not displayed during the experiment task. Also, bottom three traces represent the target path (green line) and the tracer path (black line) on each axis for the circular tracking movements on time series.

Because the height and arm length of each subject were different, the initial position of the target was calibrated before the experiments. In other words, as shown in Fig. 5A, the display position of the target was calibrated for each subject. Especially, the looking-at position of eye-convergence was accordingly set to the target position for each subject. Subjects were asked to sit straight on a fixed chair and (1) hold the controller to their chin; (2) stretch their arm forward without moving their body. The initial coordinates *P*_*x*_, *P*_*y*_, and *P*_*z*_ of the target were calculated as follows:

$${P}_{y}={S}_{y}+0.15$$

$${P}_{x}={S}_{x}$$

$${P}_{z}={T}_{z}-({T}_{z}-{S}_{z})\times 0.2$$

where *S*_*x*_, *S*_*y*_, and *S*_*z*_ are the coordinates for x-axis [m], y-axis [m], z-axis [m] of the controller in the position of subjects’chin to origin O. *T*_*z*_ is coordinates of the controller when subjects were holding their arm forward. In particular, 0.2 in *P*_*z*_ was set for the working space where to ensure no collision between the target and the HMD during the experiment. 0.15 (15 cm) in *P*_*y*_ was set as the radius of the circular movement. The two constants were set according to a preliminary test which to ensure the subject a safe and comfortable working space. The calibration allowed the experiments were carried out under a normalized condition. Beginning and ending sounds were played in each trial. Positions of the tracer and the target were recorded from the start to 1 second after each trial.

### Procedure

In this study, we performed an experiment to evaluate three-dimensional visoumotor control quantitatively using circular tracking movements on the frontal and the sagittal planes of the body in the VR space (Fig. 5B and C). Subjects were seated on a chair built for the experiment and wore a HMD. Whether stereoscopic vision can be properly perceived was confirmed orally before the experiment.

For the participants who couldn’t correctly perceive the 3D objects, inter ocular distance for each subject was accordingly adjusted. The inter ocular distance can be set in the Vive HMD function. In this study, the initial inter ocular distance was set to 64 mm according to the average value of Japanese male who were our experiment participants. In other words, the 3D display quality was confirmed for every subject before each experiment.

Subjects were asked to hold the controller by their dominant hand. Calibration was then performed to locate the initial position of the target. The target moved at the speed of 0.25 Hz along the orbit after a three-second count down with sound effect. Subjects were asked to adjust the tracer to the position of the target during the countdown and then perform circular tracking movement. As shown in Fig. 5B and C, the target stopped after three loops. One trial finished with a sound effect after the target stopped for one second. Four trials were performed for frontal plane (*ROT(0)* condition in Fig. 5B) and sagittal plane (*ROT(90)* condition in Fig. 5C) respectively. Furthermore, circular tracking movements under binocular vision and monocular vision were also investigated for each condition. The subject’s one eye was physically closed by an eye patch in the monocular condition.

Therefore, for one subject, 16 trials were carried out in the experiment. The first trial was served as exercise and was excluded from analysis.

### Statistical test

Group difference (significance test) was assessed by the paired-sample Student’s t-test (ttest function in the statistics toolbox of Matlab Ver. 7.14.0.739(R2012a).
